# Using the West Midlands CONCERT to characterise regional incidence of acute-onset post cataract surgery endophthalmitis

**DOI:** 10.1038/s41433-020-01158-6

**Published:** 2020-09-01

**Authors:** George Moussa, Hetvi Bhatt, Ian Reekie, Gibran Butt, Aaron Ng, Richard Blanch, The West Midlands CONCERT, William Fusi-Rubiano, Jalil Al-Ibrahim, Michael Quinlan, Jasvir Virdee, Sarah Dawson, Amit Patel, Liying Low, Fizza Mushtaq, Seena Nambiar, Claire Routledge, Yit Yang, Robert J. Barry, Michael Burdon, Ankur Barua, Ian De Silva, Jesse Panthagani, Madyan Qureshi, Anupama Pherwani, Mark Sigona, George Morphis, Saaeha Rauz

**Affiliations:** 1 Birmingham and Midland Eye Centre, Birmingham, UK; 2 University Hospitals of North Midlands, Stoke-on-Trent, UK; 3 Academic Unit of Ophthalmology, University of Birmingham, Birmingham, UK; 4 Academic Unit of Military Surgery and Trauma, Royal Centre for Defence Medicine, Birmingham, UK; 5 University Hospitals Birmingham, Birmingham, UK; 6 Birmingham and Midland Eye Centre, Sandwell and West Birmingham Hospitals NHS Trust, Birmingham, UK; 7 Russell’s Hall Hospital, The Dudley Group NHS Foundation Trust, Dudley, UK; 8 Heart of England Foundation Trust, now Heartlands, Good Hope and Solihull (HGS), University Hospitals Birmingham NHS Foundation Trust, Birmingham, UK; 9 New Cross Hospital, The Royal Wolverhampton NHS Trust, Wolverhampton, UK; 10 Queen Elizabeth Hospital Birmingham (QEHB), University Hospitals Birmingham NHS Foundation Trust, Birmingham, UK; 11 University Hospital Coventry And Warwickshire, University Hospitals Coventry and Warwickshire NHS Trust, Coventry, UK; 12 Royal Stoke University Hospital, University Hospitals of North Midlands, Stoke-on-Trent, UK; 13 Hereford County Hospital, Wye Valley NHS Trust, Hereford, UK

**Keywords:** Epidemiology, Eye diseases

## Abstract

**Background:**

Whilst research and innovation is embedded within the UK’s National Health Service (NHS) constitution, Doctors-in-training have little opportunity to contribute to designing, leading and recruiting into clinical trials or cohort studies. We formed the *West Midlands* *Collaborative* *Ophthalmology* *Network for* *Clinical* *Effectiveness &* *Research by* *Trainees* (The West Midlands CONCERT) and undertook a characterisation of post cataract surgery endophthalmitis as a proof-of-concept study to test the feasibility of the CONCERT model.

**Methods:**

Doctors-in-training formed a collaborative working group to test the concept of delivering a pan-regional clinical effectiveness study across multiple hospital sites by performing retrospective analyses of post cataract endophthalmitis over a 6-year period.

**Results:**

Overall, 157,653 cataract surgeries were performed by participating centres accredited to deliver the *Royal College of Ophthalmologists* training curriculum. Thirty-eight cases of post cataract endophthalmitis were identified, giving an incidence of 2.41 per 10,000 cases (0.0241%). A further 15 endophthalmitis cases presented who had surgery in non-training centres, giving a total of 53 cases. The most common organisms were *S. epidermidis* (14 (51.9%)) and *P. aeruginosa* (5 (18.5%)). Anterior-chamber and vitreous sampling yielded positive culture in 33.3% (6/18) and 50.9% (27/53), respectively. At 6 months follow-up, 19 (51.4%) patients achieved visual acuities of ≤0.5 LogMAR. Repeat intravitreal injections (11 (20.8%)) and vitrectomy (*n* = 22 (41.5%)) were not associated with better outcomes.

**Conclusions:**

Using post cataract endophthalmitis as a pilot cohort, this study highlights the feasibility of using the CONCERT model for studies across multiple sites. A UK-CONCERT could provide a powerful infrastructure enabling characterisation of patient cohorts and a platform for high-quality interventional studies, improving patient care.

## Introduction

The Health and Social Care Act 2012 states that research and innovation is embedded into the National Health Service (NHS) constitution [1]; Doctors-in-training are rarely involved in designing, leading and recruiting in studies. In the United Kingdom, Health Education England (HEE), NHS Education for Scotland (NES), Health Education and Improvement for Wales (HEIW) and Northern Ireland Medical and Dental Training Agency (NIMDTA) are responsible for implementing specialty training in accordance with General Medical Council approved specialty curricula. The Royal College of Ophthalmologists (RCOphth) is responsible for setting the curriculum and standards for ophthalmic specialist training, spanning seven years, delivered through nineteen local units—13 HEE, 4 NES, 1 HEIW and 1 NIMDTA (Supplementary Fig. 1). HEE West Midlands (WM) hosts one of the largest Postgraduate Schools of Ophthalmology with 60 trainees (9% of UK trainee ophthalmologists), including 2 medical ophthalmology trainees, distributed across 14 out of a potential 19 hospital sites across the region [2]. The WM has an area of 13,000 square kilometres with a population of 5.75 million (11% of the UK total) making it one of the most densely populated regions in the country.

The National Institute for Health Research offers an Integrated Academic Training (IAT) Programme to support individuals to gain research experience as part of their clinical training in the form of Academic Clinical Fellowships (ACFs) and Clinical Lectureships (CL). A highly competitive recruitment programme enables trainees to integrate research time within their weekly timetable to develop academic skills and research proposals for higher degrees (ACFs), or they establish themselves as clinician scientists following award of a higher degree (CL). Trainees who are not part of the IAT gain experience in research methods and clinical effectiveness projects within time dedicated to research and audit within their weekly timetable (~4 h per week) with additional time garnered through study leave (maximum 5 days per year). Due to the geographical rotational nature of the training programme delivering high-impact quality research for non-IAT trainees is limited as the ability to complete long-term meaningful projects is compromised.

Taking precedent from a number of successful trainee networks such as *Research & Audit Federation of Trainees* (RAFT, a national body of trainee anaesthetists), and *West Midlands Research Collaborative* (WMRC) the surgical trainee network that delivered the *Reduction Of Surgical Site Infection using a Novel Intervention* (ROSSINI) Trial [3], trainees from HEEWM Postgraduate School of Ophthalmology formed a network to work collaboratively to deliver pan-regional clinical effectiveness studies, retrospective and prospective interventional research. Designated the *West Midlands Collaborative Ophthalmology Network for Clinical Effectiveness & Research by Trainees* (The West Midlands CONCERT), endophthalmitis following cataract surgery was chosen by The CONCERT as a pilot study to test the feasibility of gathering data across multiple hospital sites.

Cataract Surgery is the most common surgical procedure undertaken in the NHS, with 400,000 operations performed per annum in England [4]. A sight-restoring procedure, the most feared complication is post-operative endophthalmitis which is sight threatening. According to the Endophthalmitis Vitrectomy Study Group, only 33% of patients achieve a visual acuity (VA) of 6/12 (LogMAR equivalent 0.3) or better [5, 6]. The incidence of endophthalmitis in a meta-analysis predominantly analysing published data from the UK and USA declined from 0.327% in the period 1970–1980 to 0.158% in the period 1990–2000 and then increased to 0.265% for the period 2000–2003, postulated to be due to increased practice of sutureless clear corneal incisions [5]. This frequency is substantially higher than the 0.033–0.11% reported in large tertiary referral centres and small to medium more rural hospitals in China between 2011 and 2013, respectively [7]. This difference was found to be related to posterior-capsule rupture rate, the use of povidone iodine and intra-cameral (IC) antibiotic use.

Management of endophthalmitis is resource and time-intensive [8]. The standard of investigation and treatment is immediate aqueous and vitreous sampling followed by intravitreal injection of broad-spectrum antibiotics [9]. There is no standardised guidance on the choice of intraoperative antibiotic prophylaxis for cataract surgery and this differs between centres and between surgeons [10]. Within the WM for example, a combination of IC or subconjunctival (SC) cefuroxime and/or gentamicin injection with or without corticosteroid, followed by post-operative topical eye drop antibiotics for varying periods of time is used. The national cataract audit has provided a defining incidence of post cataract endophthalmitis in the UK, 43/145,868 (0.03%) derived from data provided by participating centres between August 2007 and November 2010. There are limited data in the UK on whether best endophthalmitis treatment involves a single intravitreal injection or sequential intravitreal injections of antibiotics a few days apart, as advocated by recent studies [11, 12].

In this proof-of-concept feasibility study, the WM-CONCERT was established to demonstrate collaborative data assimilation for the characterisation of post cataract endophthalmitis: its incidence, nature of samples taken, microbiological yield, intraoperative antibiotics administered, prevalence of antibiotic resistance and clinical outcomes including VA, using a large cohort of cataract surgeries performed over a 6-year period by ophthalmology units across the WM region.

## Methods

### Establishing the collaborative network and data handling

Doctors-in-training (Residents) from the HEEWM formed a collaborative working group by inviting NHS Trusts accredited to deliver the *Royal College of Ophthalmologists* training curriculum to join the West Midlands CONCERT network. Each participating hospital was assigned a lead doctor-in-training and consultant. For larger hospital consortia comprising several hospital sites, a lead consultant and trainee were identified for each of the training hospital sites. In order to test the feasibility of delivering a pan-regional clinical effectiveness programme, the project protocol was approved by the Clinical Effectiveness Department within each Hospital Trust. Data were captured in validated study-site record forms with anonymised patient and centre unique identifiers. Merging and cleaning of data from each site were handled in accordance to the tenets of General Data Protection Regulations. The project data monitoring, steering and writing committee comprising the overall Lead Consultant for the WM-CONCERT (SR) and six trainees (GM, HB, IR, GB, AN and RB) met quarterly to assess progress, milestones, risks, mitigation steps, interim analyses and critical endpoints.

### Testing the CONCERT Model: prevalence of post cataract surgery endophthalmitis

In order to test the feasibility of the CONCERT model, the steering committee considered high volume of cases and rare intervention or complication as two essential inclusion criteria for its inaugural retrospective pilot project. A cohort study to analyse adult cases of post cataract endophthalmitis over a 6-year study period (Jan 2010–Dec 2015) fulfilled these criteria. A wide period of interest was required in order to capture a meaningful number of cases of what is a rare surgical complication. Surgical technique did not evolve substantially during this time. Data collection was between February and December 2018: this enabled adequate follow-up data on clinical outcomes, and report best final visual outcome.

Thirteen NHS Trusts (hubs) served 23 spoke hospitals across the WM performing cataract surgeries. Eleven units were excluded from the study either because they were non-training private healthcare providers (*n* = 6), non-accredited training units (*n* = 4) and a dedicated Children’s Hospital (*n* = 1 (study cohort >18 years age)). A further two training units elected not to participate. The remaining 11 ophthalmology training units provided total numbers of cataract surgery performed during the study period.

### Microbiology data and visual outcomes analysis

Each participating hospital was assigned a lead doctor-in-training and consultant for the project. To ensure as complete capture as possible of endophthalmitis cases, we explored various methods to identify previous cases. These included:

- Official trust audit data of endophthalmitis cases.
- Microbiology database of all vitreous and aqueous samples analysed.
- Local endophthalmitis logbooks when available.
- Discussion with consultants regarding their post-operative endophthalmitis patients

The doctor-in-training contacted the local microbiology department, and cases were identified by screening all aqueous and vitreous samples passing through microbiology departments. Cases of post-operative endophthalmitis other than cataract surgery, endogenous endophthalmitis and pan-uveitis cases were excluded. Clinical data were extracted from electronic and paper records. Of these four mechanisms to capture endophthalmitis cases, we found the microbiology database the most complete and captured all cases identified by other methods. As all presumed cases of post-operative endophthalmitis would have vitreous sampling ± aqueous sampling as part of the work up, it is possible to have a high level of confidence that all cases of endophthalmitis presenting to the hospitals participating in the study were recorded.

The CONCERT collected data from the surgical unit that performed the cataract surgery and the unit to which the patient presented, including the acute presentation, intraoperative details, initial and subsequent treatments together with investigations undertaken and microbiology results. Several units did not have a dedicated emergency service for Ophthalmology emergencies and referred cases to the regional tertiary centre through a hub-and-spoke arrangement for the provision of emergency care (Supplementary Fig. 2A). For such patients, coordination by the CONCERT was critical in obtaining pre-operative and intraoperative data from the spoke surgical unit and aligning it with data relating to the endophthalmitis episode at the tertiary referral centre (Supplementary Fig. 2A, B). These included units that do not have trainees. Microbiology, presentation, and treatment data was available for 53 cases included in the analysis that presented to tertiary hub referral centres.

### Statistical analysis

Each participating centre recorded source data in a validated study-site spreadsheet (Supplementary File 1) generated in Excel^®^ (Microsoft Corporation, Redmond, WA). Data from each study-site spreadsheet were imported into a study master-spreadsheet which amalgamated data from all participating sites and enabled both preliminary descriptive analyses as well as data export to IBM SPSS Statistics for Windows, Version 25.0 (IBM Corp, Armonk NY) for statistical analysis, significance defined as *p* < 0.05. Prior to analysis, continuous variables were assessed using the Shapiro–Wilk test, and found not to be normally distributed. Hence, data are primarily reported as medians and interquartile ranges (IQRs) throughout. A Levene analysis showed no significant difference in variance in groups being compared. Mann–Whitney *U* and Wilcoxon signed rank tests were used for un-paired VA data and two-paired VA data, respectively. Fisher exact test was used for nominal variables. Bonferroni correction is applied for multiple statistical analysis. Best corrected VA was used; where records were in Snellen, conversion to LogMAR was undertaken. For lower VA, corresponding to count fingers (CF), hand movements (HM), perception of light (PL) and no PL (NPL) were substituted with 2.10, 2.40, 2.70 and 3.00 LogMAR, respectively, in keeping with previous publications from the National Ophthalmology Database (NOD) group [13].

## Results

### Prevalence and patients

Overall, 157,653 cataract surgeries were performed by participating centres during the study period. A breakdown of which units were included is available in Supplementary Figs. 2A, B. Thirty-eight cases of post cataract endophthalmitis were identified, giving an incidence of 2.41 in 10,000 cases (0.0241%). A further 15 endophthalmitis cases presented who had surgery performed in non-training centres, giving a total of 53 cases.

Patients presented at median 6.5 (IQR 3.4–15.0) days. One patient presented at 744 days, having been treated for chronic non-resolving and progressive pan-uveitis since surgery, later diagnosed as a chronic fungal endophthalmitis. Vitreous sampling was performed as per local hospital practice, with 21G or 23G needle immediately before administration of antibiotics. In two units, this was performed utilising a single port vitrector with antibiotics delivered at the end of the procedure. Aqueous sampling was performed utilising an insulin syringe.

### Clinical utility of culture data

During the study period, no endophthalmitis management protocol involved repeat sampling or repeat intravitreal antibiotics. Despite this, 11 (17.3%) had repeat intravitreal injections. One (1.9%) patient was reinjected without positive growth/sensitivity data, with amphotericin B for presumed fungal infection.

### Detection rates and Investigations microbiological sampling

No cases had organisms detected in aqueous or by PCR that were not also detected in vitreous. In 15 cases both aqueous and vitreous cultures were sent. In ten cases, vitreous cultures were positive, of which six also had positive aqueous cultures (*p* = 0.275, Fisher’s Exact test). In seven patients, vitreous fluid was sent for both PCR and culture. Of these seven, three patients were both PCR and vitreous culture positive demonstrating good concordance between PCR detection and vitreous culture. These results are shown in Table 1.

**Table 1:** Investigations and results.

Investigation performed	Samples sent *n* (%)	Positive yield for each investigation *n*/total (%):		
		Aqueous	Vitreous	PCR
Vitreous culture only	31 (58.5)	–	15/31 (48.4)	–
Both AC and vitreous culture	15 (28.3)	6/15 (40.0)	9/15 (60.0)	–
Vitreous culture and vitreous PCR	4 (7.6)	–	2/4 (50.0)	2/4 (50.0)
AC and vitreous culture and vitreous PCR	3 (5.7)	0/3 (0.0)	1/3 (33.3)	1/3 (33.3)
Total	53 (100.0)	6/18 (33.3)	27/53 (50.9)	3/7 (42.8)

Detection rates from vitreous, aqueous and PCR investigation.

*PCR* polymerase chain reaction, *AC* anterior chamber.

### Bacteriology

Positive cultures and antibiotic sensitivity were obtained in 27/53 (50.9%) cases (Table 1). The most common organism was *Staphylococcus epidermidis* (14 (51.9%)) followed by *Pseudomonas aeruginosa* 5 (18.5%) and one case (3.7%) each of *Candida parapsilosis*, *Serratia marcescens*, *Streptococcus* *haemolyticus*, *Serratia liquefans*, Methicillin-Resistant *Streptococcus* *aureus*, *Streptococcus ovis*, *Streptococcus pneumoniae* and *Streptococcus parasanguinis*.

### Antimicrobial use and reporting

Supplementary Table 1 shows antimicrobial/classes of antibiotic reporting of sensitivities for intravitreal injections/systemic and topical augmentation, those with clinical implication in endophthalmitis highlighted with an asterisk. The most common antibiotic used intraoperatively during cataract surgery was cefuroxime; sensitivity was reported in two cases, both of which were resistant. The most common systemic antibiotic used was ciprofloxacin used in 36 (67.9%) cases. Of the 27 patients with positive growth, ciprofloxacin sensitivity was reported in 15 (55.6%) cases, of which 6 (22.2%) were resistant. On subgroup analysis where ciprofloxacin sensitivity was reported, resistance was found in 6/15 (40.0%). For *S. epidermidis*, resistance to ciprofloxacin was demonstrated in 5/9 (55.6%) cases. One case of *S. epidermidis*, demonstrated vancomycin resistance, and another had intermediate sensitivity to vancomycin. Both cases were gentamicin sensitive, and one ciprofloxacin resistant. Cephalosporin sensitivity was not reported in either of those cases.

### Visual acuity (VA)

Apart from one case, all patients presented prior to their first follow-up visit and as such no post-surgery objective VA was available. Patients presented with a subjective reduction of vision from post-surgery. Six-months VA was available in 37/53 (69.8%) patients. Missing data were due to patients lost to follow-up, incomplete transference of data to electronic records, follow-up in private clinics/non-training hospitals and 2 (3.7%) patients deceased during the period.

**Fig. 1:**
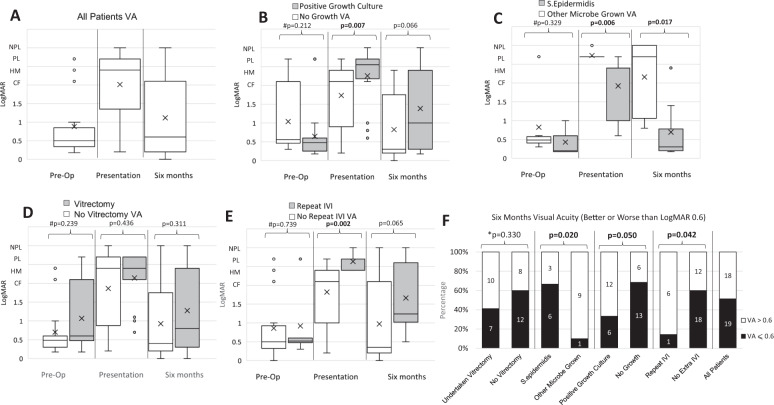
Visual outcomes and subgroup analysis, Box and Whisker plot. ‘X’ denotes mean. Hash symbol ‘#’ denotes Mann–Whitney *U* test; plus symbol ‘+’ denotes Wilcoxon Signed Rank Test. An asterisk ‘*’ denotes Fishers Exact test. Bonferroni critical *p* value 0.017 (**a**–**e**), 0.05 (**f**). Statistical significance in bold. **a** Representation of all patients’ visual acuity. **b** Difference in visual acuity between patients that had a positive culture on vitreous biopsy compared to no growth. **c** Vision in patients that had *S. epidermidis* growth vs. other microbes. **d** Vision in patients that underwent vitrectomy vs. no vitrectomy. No significance was seen at all time frames. **e** Vision in patients that had additional intravitreal injection (IVI) of antibiotic vs. those that did not. **f** Subgroup analysis (Fisher’s Exact test) at 6 months visual acuity of patients achieving 0.6 LogMAR or better visual acuity.

**Fig. 2:**
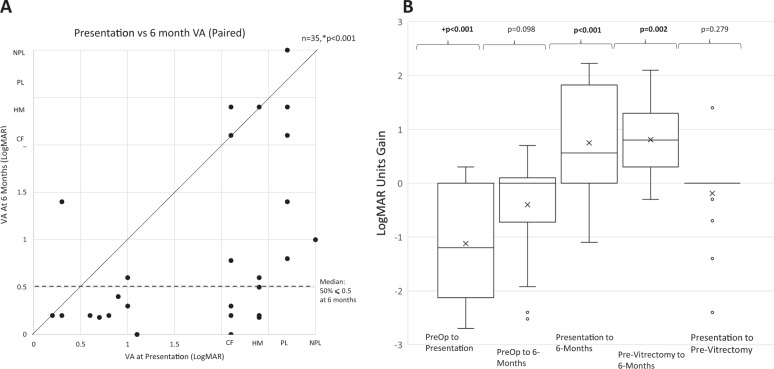
Comparing paired visual acuity of patients at different time points. *Wilcoxon Signed Rank Test, Bonferroni critical *p* value 0.05 (**a**), 0.01 (**b**). Statistical significance in bold. **a** Scatterplot of paired presentation vs. 6-month VA. Median VA 0.5: 50% of patients achieved vision equal or better than 0.5 LogMAR. **b** Box and Whisker plot of paired VA LogMAR units gain at different time points. ‘X’ denotes mean.

VA for the whole cohort (Fig. 1a, f) and for subgroup analysis (Fig. 1b–f) are reported across different time points. Paired VA data at different time points are reported in Fig. 2a, b. Positive vitreous culture (Fig. 1b, f) and the need for repeat intravitreal antibiotics (IVI) (Fig. 1e, f) had worse visual outcomes at 6 months. A growth of *S. epidermidis* infections demonstrated better VA outcomes compared to other microbes (Fig. 1c, f).

Patients requiring vitrectomy had a worse pre-operative VA (Fig. 1d) with vitrectomy being performed in 22 (41.5%) of patients (7 (41.2%) <24 h; 15 (70.5%) <2 weeks). At 6 months, whilst 50% of all patients achieved VA ≤ 0.5 (Fig. 2a), vitrectomy was not associated with better visual outcomes (Fig. 1d, f).

## Discussion

This study highlights that the CONCERT model has the potential to deliver outcomes from a pan-regional data collection initiative across multiple sites. Taking as a precedent RAFT and WMRC, the WM-CONCERT has demonstrated an ability to bring together individual hospitals, share and analyse data with the ultimate aim of standardising patient care across the region and to provide an infrastructure for high powered multicentre interventional trials. Working within the network, the CONCERT was able to simultaneously access patient records and data across 15 different sites and feed into a master-file to efficiently generate descriptive and more meaningful statistics than would be possible if analysing data from a single centre alone.

Although there are many advantages to a collaborative network such as the CONCERT, there were two key hurdles that were encountered. First, the Excel spreadsheet was useful for this feasibility study for local site data entry in the context of low sample size (such as endophthalmitis cases) as it allowed multicentre site files to be exported and merged into a trial master excel file. Nevertheless, usability is limited for larger sample sizes and for complex randomised controlled studies across many sites regionally or nationally. The second hurdle related to the curriculum-driven rotation of Trainees across the region to various sites. Delays in handover by the outgoing trainees to the incoming trainees led to periods of natural suspension before data collection resumed. To overcome this, the CONCERT is integrating browser-based technology in its methodology using Research Electronic Data Capture (REDCap^®^ Vanderbilt University), a software workflow system for designing clinical and translational research databases. This is a centralised web-based database with mobile device application tokens for lever date entry that will significantly simplify the data collection process as it enables direct continuity between patients that were transferred across different trusts, and between rotating trainees. This system is being used widely to enhance consortia and the delivery of collaborative research through international research networks and has recently been adopted by WMSRC. Embedding a browser-based data workflow package will provide a sound infrastructure for the WM-CONCERT to work with other regional CONCERTs across the country to deliver a UK-CONCERT to support high-impact investigator led clinical trials.

The CONCERT data for this feasibility study provided an overall rate of post-phacoemulsification endophthalmitis across 11 treatment centres as 2.41 per 10,000 cases. Table 2 [13–20] presents the incidence of endophthalmitis found in this study in comparison with other studies of post-operative endophthalmitis. As a multicentre study, we included a broader range of surgical techniques and peri- and post-operative antimicrobial prophylaxis regimes than single-centre studies and therefore provide a more representative estimate of overall incidence. In all the studies, the large majority of cataract operations were carried out by phacoemulsification. The UK NOD audit, reported by Donachie et al., is included to give a national perspective across England and Wales. It is notable however that 22.7% of operations lacked recorded post-op data in this cohort, and that the rate of endophthalmitis was not a primary outcome measure for the audit [13]. A great advantage of a national database is that it can give a nationwide view of surgical complications, and the NOD captured ~20% of cataract surgeries performed during its data collection period and report 65% reporting of post-operative VA data. As the NOD primary aim was to report risk adjusted rates for PCR and VA loss in cataract surgery, there was no microbiology data reported for endophthalmitis cases [13]. In our study, we estimate that we captured 65% of cataract surgeries performed in the region. This was achieved by using NOD report 4 (appendix 7) to estimate number of surgeries performed per year, in units we did not have surgical numbers for [21]. We report microbiology sensitivities in 53 (100%) of cases and 6-month VA data in 37 (70%) of cases.

**Table 2:** Endophthalmitis rates in reported adult case series.

Study	Time Period	Location	Number of Operations	Incidence of endophthalmitis *n* (%)	Comments
This study (2019)	2010–2015	West Midlands, UK	157,653	37 (0.025%)	Retrospective cohort
Donachie et al. (2016) [13]	2014***–***2015	UK	75,827	14 (0.018%)	National Ophthalmology Database (NOD) Audit Retrospective, cross sectional study
Inoue et al. [14]	2012–2013	Japan	52,983	13 (0.025%)	Prospective study of cases presenting up to 8 weeks post-op
Creuzot-Garcher et al. [15]	2005–2014	France	6,371,242	6668 (0.105%)	Retrospective cohort, cases up to 6 weeks post-op
Nam et al. [16]	2006–2009	Gyeongsangnam-do and Pusan city, South Korea	192,747	71 (0.037%)	Retrospective, cross-sectional study
Lundström et al. [17]	2002–2010	Sweden	692,786	244 (0.035%)	Retrospective cohort
Hatch et al. [18]	2002–2006	ON, Canada	442,177	617 (0.140%)	Retrospective cohort
Montan et al. [19]	1998	Sweden	54,666	58 (0.100%)	Prospective study, follow-up to 2 years post-op
Mollan et al. [20]	1996–2004	Birmingham, UK	101,920	105 (0.103%)	Retrospective cohort

Multicentre studies of incidence. All figures reported post cataract endophthalmitis. Creuzot-Garcher et al. [15] specify all cases are phacoemulsification. Inoue et al. [14], were 99.4% phaco, 0.5% ECCE, 0.1% ICCE. The other series do not explicitly specify operating technique, but majority of cases are presumed phacoemulsification from the years of data collection. No one method of antibiotic prophylaxis was used; generally, a mixture of intra-cameral, subconjunctival and topical depending on the operating surgeon/institution.

Our study however has the considerable advantage of associated microbiology data with cases of endophthalmitis, as well as including a greater number of cataract operations due to the longer window for data collection.

There is an apparent reduction in the rate of endophthalmitis following phacoemulsification surgery over time (Table 2), perhaps due to increasing use of IC cefuroxime prophylaxis [15]. Mollan et al. reported post cataract endophthalmitis between 1996 and 2004 from Birmingham Midland Eye Centre (UK). This is a regional tertiary referral centre also included in our study. In Mollan et al.’s data set endophthalmitis case referrals originated from 13 operating suites, and they reported an incidence of 0.099% (i.e. almost 1 per 1000 cases) over an 8-year period [20]. Extracting data from the CONCERT dataset for the same participating sites, we report an incidence of 0.0179% (15 out of 83,867 cases i.e. 1.7 per 10,000 cases over a 6-year period) demonstrating a substantial reduction in incidence. Being a retrospective case series, some of the analyses were restricted by this inherent study design limitation. For example, we could not compare SC to IC antibiotics rate of endophthalmitis; even within units that a principle change in practice from SC to IC took place, interdepartmental variability in practice, between clinicians, persisted.

We present 53 cases of post-phacoemulsification endophthalmitis, of which 26 (50.9%) yielded positive microbial culture from either vitreous culture alone or vitreous plus aqueous culture. This represents the largest cohort of patients evaluated in the UK to date. There was no case in this series where an aqueous sample gave a positive culture and a vitreous sample did not. Conversely, in four cases a positive vitreous culture was accompanied by a negative aqueous culture from the same patient, suggesting that aqueous culture has poorer sensitivity. These findings are in keeping with both studies in animal models [22] and those previously reported in clinical studies showing aqueous tap culture-positive rates in 20–37.7% of clinically diagnosed endophthalmitis cases compared to 48.8–74% of vitreous samples [23, 24]. Despite overall greater sensitivity of vitreous cultures, some studies report the occasional aqueous sample culture showing pathogenic microorganism growth (rather than contaminant) where a corresponding vitreous sample culture is negative [25, 26].

The results presented here support the use of vitreous tap culture in clinically identified cases of post-operative endophthalmitis. Although there was a 49.1% culture-negative rate provided on average 72 h after sampling, indicating the need for more specific rapid molecular diagnostics or better optimisation of laboratory culture protocols to yield improved positive identification of causative organisms. In addition, while we recognise the relatively small numbers of aqueous samples taken in this study, consideration should be given to whether aqueous taps are required in all cases of endophthalmitis given the low likelihood of aqueous sampling providing additional useful diagnostic information to vitreous tap. While VA was significantly better at 6 months follow-up compared to presentation; pre-operative VA was significantly better than both presentation and final VA. Post-surgical VA was not available as not all patients had had their first post-operative visit. Vitrectomy had no effect on visual outcome, although culture-positive cases seemed to indicate poorer prognosis.

Post-operative endophthalmitis is thought to be most commonly caused by the intra-ocular inoculation of the patient’s bacterial flora [27]. Gram-positive organisms were the most prevalent in the cohort presented here, *S. epidermidis* was identified in 53.8% of culture isolates. The spectrum of causative organisms in this study is largely consistent with previously published data [28–30]. Geographical variability is recognised, with one study from Taiwan demonstrating *Enterococcus faecalis* as marginally more common bacterium than *S. epidermidis* [31]. Fungal species are rare causes of post-surgical endophthalmitis in temperate regions [28, 29, 32]. A recent study on virulence of *S. epidermidis* by Chiquet et al. [33]. demonstrated that strains that caused endophthalmitis differed from those that did not. More virulent strains had an increased likelihood of carrying genes coding for adhesion and biofilm formation and also had increased antibiotic resistance. However, numerous studies have shown that patients with *S. epidermidis* endophthalmitis have better visual outcomes compared to those patients with *Enterococcus* [31], *S. aureus* and gram-negative bacteria [34]. Our study confirmed this; patients with *S. epidermidis* endophthalmitis have significantly better presenting visual acuities and better 6-month post-presentation VA compared to those that grew other micro-organisms.

Initial treatment for endophthalmitis is empirical. Typical antimicrobial agents used are vancomycin providing excellent Gram-positive coverage, and ceftazidime or amikacin for Gram-negative coverage. Antibiotic sensitivity data from our cohort shows most Gram-positive organisms were sensitive to vancomycin; however, one isolate of *S. epidermidis* was resistant. Many other published studies suggest almost universal vancomycin sensitivity amongst Gram-positive bacteria [28, 31, 32], with only very sporadic resistant cases [29, 30]. A recent retrospective study of endophthalmitis cases from a patient population geographically close to our cohort showed 100% Gram-positive bacterial sensitivity to vancomycin [35]. The potentially devastating impact of vancomycin resistance in these organisms should prompt careful monitoring of bacterial resistance patterns. Cefuroxime is the most common antibiotic used intraoperatively in our region, but sensitivity was reported in just two cases with resistance in both, highlighting a potential future problem with resistance. The most common systemic antibiotic used for the management of endophthalmitis in this cohort was ciprofloxacin (66.7% of cases). From our data, where ciprofloxacin sensitivity was reported, there are high resistance rates for a first line antibiotic (40.0%). Systemic moxifloxacin has been shown to have ocular penetration than ciprofloxacin, potentially underlying its great efficacy [36].

There was little standardisation of microbiological sensitivity reporting for ophthalmic infections in this cohort, with vancomycin, cephalosporin and aminoglycoside sensitivity reported in just (51.8%), 5 (18.5%) and 20 (74.1%) of cases respectively (Supplementary Table 1). Given the standardisation of treatment, especially initial therapy, it would be beneficial for microbiology laboratories to standardise their sensitivity testing for intra-ocular culture samples with appropriate antibiotic panels. In particular, testing for cefuroxime sensitivity should be undertaken routinely given its widespread use as prophylaxis. Classically microscopy, culture and sensitivity has been the gold-standard in identifying pathogens in endophthalmitis. However, this presents challenges in an ophthalmology setting, with relatively low sensitivities from aqueous and vitreous samples and the time-consuming nature of these investigations delaying more tailored treatment. There has been increasing evidence that real-time PCR may be beneficial when performed alongside more established methods. Therese et al. [37] demonstrated that positive specimens increased by 29.3% when PCR was used in addition to microscopy and culture. In addition, a study by Tarai et al. looking at fungal endophthalmitis showed PCR sensitivity of 50% and specificity of 100% [38]. Many studies have also demonstrated that PCR is a useful tool when cultures are negative (Table 3) [39–43]. There has however been a reported 14% false positive rate of PCR [44]. This may limit its use to cases where culture is negative, and another method is required to identify pathogens. In our cohort, PCR had a comparable sensitivity to vitreous culture; seven cases had both investigations and results were concordant in all seven.

**Table 3:** Previous studies of polymerase chain reaction (PCR) in post cataract endophthalmitis.

Study	Sample size	Type of sample	Culture +ve PCR −ve	Culture −ve PCR +ve	Culture and PCR +ve	Culture and PCR −ve
This Study	7	Vitreous	0	0	3	4
Joseph et al. [39]	64	Vitreous	1	19	18	26
Lohman et al. [40]	25	Aqueous	0	21	0	4
Lohman et al. [40]	25	Vitreous	0	17	6	2
Lohman et all. [41]	16	Aqueous	0	15	1	0
Lohman et all. [41]	16	Vitreous	0	7	7	2
Chiquet et al. [42]	30	Aqueous	2	9	6	9
Sowmya et al. 2009*	72	Aqueous	3	32	24	13
Sownya et al. 2009* [43]	72	Vitreous	2	40	25	5

An asterisk denotes post cataract, post traumatic and endogenous endophthalmitis.

Overall, we report a post-phacoemulsification endophthalmitis incidence comparable to previous studies. Microbial culture remains key to organism identification, and culture from vitreous tap may be more sensitive than aqueous tap. Gram-positive bacteria were the main causative organisms. Resistance to commonly used antibiotics, particularly vancomycin and ciprofloxacin, are of concern and it is vital that ophthalmology departments regularly audit sensitivity data from infecting organisms to determine the most appropriate empirical antibiotics for local bacterial antibiotic resistance patterns. Close collaboration between ophthalmic and microbiology departments may enable use of standardised ophthalmic antibiotic sensitivity reporting. Analysing our data geographically and chronologically did not reveal any geographic clusters or changing bacterial sensitivity over time. A UK wide national CONCERT may be required to demonstrate this.

In summary, the CONCERT model has facilitated the delivery of a retrospective region-wide descriptive study of post-surgical endophthalmitis. We provide a regional incidence of the most feared complication of cataract surgery in the largest published UK dataset to date. Progression to prospective longitudinal phenotyping studies using a web-based and mobile device data capture system is currently underway. The WM-CONCERT, working in partnership with other regional CONCERTS, has the scope to develop and deliver collaborative projects of complex design. A UK-CONCERT could provide an infrastructure to characterise national cohorts and implement interventional studies of novel therapeutic technologies, to standardise and deliver better clinical outcomes and improve patient care.

### Summary

#### What was known before

- Management of endophthalmitis is resource and time-intensive, and there is no standardised guidance on the choice of intraoperative antibiotic prophylaxis for cataract surgery.
- Regional variation exists between centres and between surgeons, in methods of acquiring microbiological samples and use of microbiology sensitivity and resistance panels.

#### What this study adds

- Ophthalmology doctors-in-training formed the West Midlands Collaborative Ophthalmology Network for Clinical Effectiveness & Research by Trainees (The West Midlands CONCERT), working across the region to produce the largest published UK dataset of post cataract endophthalmitis and showing an incidence of 2.41/10,000 surgeries (0.0241%).
- A collaborative approach to data gathering means our multicentre CONCERT study estimate of the incidence of endophthalmitis following cataract surgery is likely to be representative of the wider population.
- Partnering with other regional CONCERTS and forming a UK-CONCERT has the potential to deliver impactful projects including phenotyping of national cohorts and undertaking complex interventional studies with the aim of improving patient care.

## Supplementary information

Supplementary Figure 1

Supplementary Figure 2A

Supplementary Figure 2B

Supplementary Table 1

Supplementary File 1
