# Supplementary material for: Using the West Midlands CONCERT to characterise regional incidence of acute-onset post cataract surgery endophthalmitis
Source: Eye (Lond). 2020 Sep 1;35(6):1730–40. doi: 10.1038/s41433-020-01158-6 (PMC8169918; doi:10.1038/s41433-020-01158-6)
Supplement: Supplementary file 1 — Supplementary Figure 1 [file 41433_2020_1158_MOESM1_ESM.docx]

**Supplementary Figure 1**


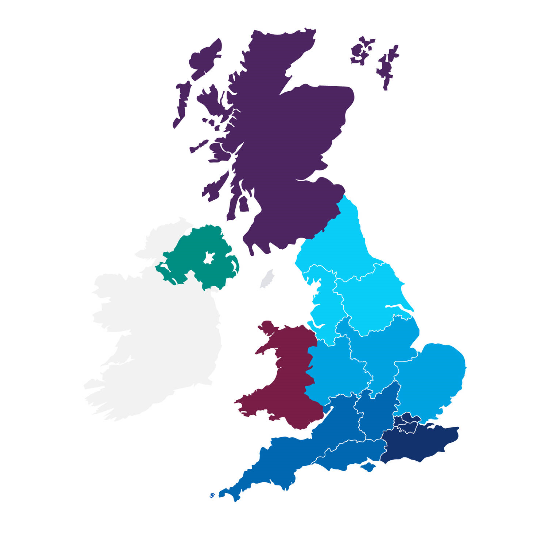

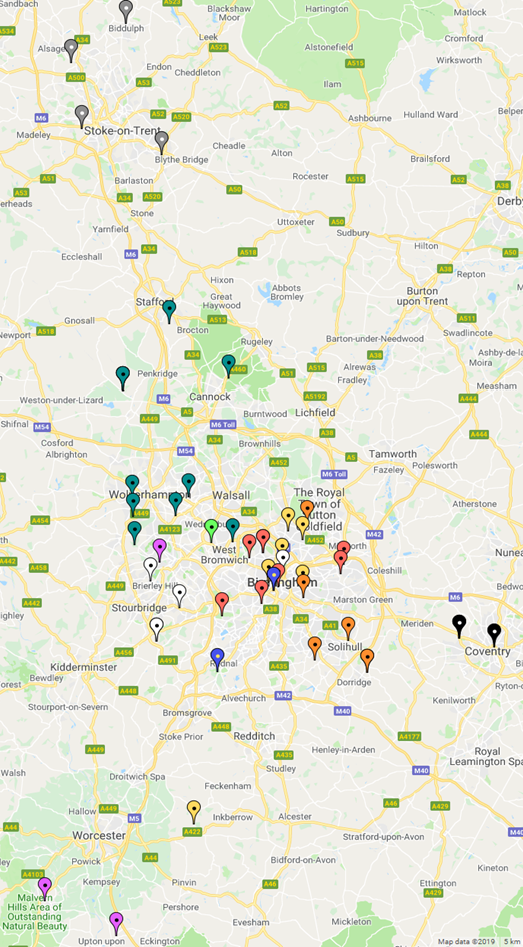


**B**

**A**

|  | Royal Stoke University Hospital |
| --- | --- |
|  | New Cross + Cannock Chase Hospital |
|  | Other |
|  | Birmingham and Midland Eye Centre |
|  | Solihull Hospital |
|  | University Hospital of Coventry and Warwickshire |
|  | Worcester Royal, Kidderminster + Alexandra Hospital |
|  | Russell’s Hall Hospital, Dudley |
|  | Queen Elizabeth Hospital Birmingham |
|  | Sandwell General Hospital |

**A**) Training Across the UK: RCOphth Curriculum across 13 Health Education England (HEE), 4 NHS Education for Scotland (NES), 1 Health Education and Improvement for Wales (HEIW) and 1 Northern Ireland Medical and Dental Training Agency (NIMDTA) areas

**B**) Location of patients from hospitals across the West Midlands showing geographical distribution.
