# Supplementary material for: Using the West Midlands CONCERT to characterise regional incidence of acute-onset post cataract surgery endophthalmitis
Source: Eye (Lond). 2020 Sep 1;35(6):1730–40. doi: 10.1038/s41433-020-01158-6 (PMC8169918; doi:10.1038/s41433-020-01158-6)
Supplement: Supplementary file 2 — Supplementary Figure 2A [file 41433_2020_1158_MOESM2_ESM.docx]

**Supplementary Figure 2A:**

Main tertiary referral hubs and the spokes they serve.

White: Hospitals without trainees from the Health Education England West Midlands Postgraduate School of Ophthalmology

Green: RCOphth accredited teaching hospitals

Red: Accredited teaching hospitals where total number of cataract surgeries was not available
