# Supplementary material for: Using the West Midlands CONCERT to characterise regional incidence of acute-onset post cataract surgery endophthalmitis
Source: Eye (Lond). 2020 Sep 1;35(6):1730–40. doi: 10.1038/s41433-020-01158-6 (PMC8169918; doi:10.1038/s41433-020-01158-6)
Supplement: Supplementary file 4 — Supplementary Table 1 [file 41433_2020_1158_MOESM4_ESM.docx]

| **Antibiotic** | ***Staphylococcus*** | | ***Streptococcus*** | | ***Pseudomonas*** | | ***Serratia*** | | ***Candida*** | | **Total** | |
| --- | --- | --- | --- | --- | --- | --- | --- | --- | --- | --- | --- | --- |
|  | **Sensitive**  **n (%)** | **Resistant**  **n (%)** | **Sensitive**  **n (%)** | **Resistant**  **n (%)** | **Sensitive**  **n (%)** | **Resistant**  **n (%)** | **Sensitive**  **n (%)** | **Resistant**  **n (%)** | **Sensitive**  **n (%)** | **Resistant**  **n (%)** | **Sensitive** | **Resistant** |
| Amikacin* |  |  |  |  | 1 (20.0) |  |  |  |  |  | 1 (3.7) | 0 (0.0) |
| Amphotericin* |  |  |  |  |  |  |  |  | 1 (100.0) |  | 1 (3.7) | 0 (0.0) |
| Ceftazadime* |  |  |  |  | 2 (40.0) |  |  |  |  |  | 2 (7.4) | 0 (0.0) |
| Cefotaxime |  |  |  |  |  |  | 1 (50.0) |  |  |  | 1 (3.7) | 0 (0.0) |
| Cefuroxime* |  | 1 (6.3) |  |  |  |  |  | 1 (50.0) |  |  | 0 (0.0) | 2 (7.4) |
| Chloramphenicol* | 11 (68.8) | 1 (6.3) |  |  |  | 1 (20.0) |  |  |  |  | 11 (40.7) | 2 (7.4) |
| Ciprofloxacin* | 4 (25.0) | 6 (37.5) |  |  | 3 (60.0) |  | 2 (100.0) |  |  |  | 9 (33.3) | 6 (22.2) |
| Clindamycin | 2 (12.5) | 1 (6.3) | 1 (33.3) |  |  |  |  |  |  |  | 3 (11.1) | 1 (3.7) |
| Co-amoxiclav |  |  |  |  |  |  |  | 2 (100.0) |  |  | 0 (0.0) | 2 (7.4) |
| Daptomycin | 1 (6.3) |  |  |  |  |  |  |  |  |  | 1 (3.7) | 0 (0.0) |
| Erythromycin | 4 (25.0) | 3 (18.8) | 1 (33.3) | 1 (33.3) |  |  |  |  |  |  | 5 (18.5) | 4 (14.8) |
| Flucloxacillin | 1 (6.3) | 11 (68.8) |  |  |  |  |  |  |  |  | 1 (3.7) | 11 (40.7) |
| Fluconazole |  |  |  |  |  |  |  |  | 1 (100.0) |  | 1 (3.7) | 0 (0.0) |
| Fucidic acid | 1 (6.3) | 6 (37.5) |  |  |  |  |  |  |  |  | 1 (3.7) | 6 (22.2) |
| Gentamicin* | 13 (81.3) |  |  |  | 4 (80.0) |  | 2 (100.0) |  |  |  | 19 (70.4) | 0 (0.0) |
| Linezolid* | 3 (18.8) |  |  |  |  |  |  |  |  |  | 3 (11.1) | 0 (0.0) |
| Meropenem* |  |  |  |  | 1 (20.0) |  |  |  |  |  | 1 (3.7) | 0 (0.0) |
| Moxifloxacin* |  |  | 1 (33.3) |  |  |  |  |  |  |  | 1 (3.7) | 0 (0.0) |
| Penicillin* | 2 (12.5) | 8 (50.0) | 2 (66.7) |  |  |  |  |  |  |  | 4 (14.8) | 8 (29.6) |
| Piperacillin/tazobactam |  |  |  |  | 3 (60.0) |  |  |  |  |  | 3 (11.1) | 0 (0.0) |
| Rifampacin | 2 (12.5) | 2 (12.5) |  |  |  |  |  |  |  |  | 2 (7.4) | 2 (7.4) |
| Teicoplanin* |  | 1 (6.3) |  |  |  |  |  |  |  |  | 0 (0.0) | 1 (3.7) |
| Tetracycline | 3 (18.8) | 5 (31.3) | 1 (33.3) |  |  |  |  |  |  |  | 4 (14.8) | 5 (18.5) |
| Trimethoprim |  | 1 (6.3) |  |  |  |  | 2 (100.0) |  |  |  | 2 (7.4) | 1 (3.7) |
| Vancomycin* | 12 (75.0) | 1 (6.3) | 1 (33.3) |  |  |  |  |  |  |  | 13 (48.1) | 1 (3.7) |
| Total Infections | 16 (59.3) | | 3 (11.1) | | 5 (18.5) | | 2 (7.4) | | 1 (3.7) | | 27 (100.0) | |

**Supplementary Table 1 – Antibiotic sensitivities in culture positive cases.**

Organisms and microbiology reporting for sensitivity and resistance.

*Antimicrobials with clinical relevance to endophthalmitis treatment.
